# CRISPR/Cas9 Editing Sites Identification and Multi-Elements Association Analysis in *Camellia sinensis*

**DOI:** 10.3390/ijms242015317

**Published:** 2023-10-18

**Authors:** Haozhen Li, Kangkang Song, Bin Li, Xiaohua Zhang, Di Wang, Shaolin Dong, Long Yang

**Affiliations:** College of Plant Protection and Agricultural Big-Data Research Center, Shandong Agricultural University, Tai’an 271018, China

**Keywords:** *Camellia sinensis*, CRISPR/Cas9, G-quadruplexes, SSRs, GC content

## Abstract

CRISPR/Cas9 is an efficient genome-editing tool, and the identification of editing sites and potential influences in the *Camellia sinensis* genome have not been investigated. In this study, bioinformatics methods were used to characterise the *Camellia sinensis* genome including editing sites, simple sequence repeats (SSRs), G-quadruplexes (GQ), gene density, and their relationships. A total of 248,134,838 potential editing sites were identified in the genome, and five PAM types, AGG, TGG, CGG, GGG, and NGG, were observed, of which 66,665,912 were found to be specific, and they were present in all structural elements of the genes. The characteristic region of high GC content, GQ density, and PAM density in contrast to low gene density and SSR density was identified in the chromosomes in the joint analysis, and it was associated with secondary metabolites and amino acid biosynthesis pathways. CRISPR/Cas9, as a technology to drive crop improvement, with the identified editing sites and effector elements, provides valuable tools for functional studies and molecular breeding in *Camellia sinensis*.

## 1. Introduction

*Camellia sinensis* is an important perennial cash crop and one of the most widely consumed non-alcoholic beverages in the world, with health benefits [1]. *Camellia sinensis* enriched with secondary metabolites that provide aroma, freshness, and astringent flavour are key determination factors contributing to its quality hence, it is particularly important to explore their influencing factors. Current emerging gene editing could regulate plant traits and improve crops through selection of target loci. The study of CRISPR/Cas9, with included simple sequence repeats (SSR) and G-quadruplex affected elements, is expected to provide an opportunity for *Camellia sinensis* breeding improvement through the perspective of gene editing.

A variety of target gene-editing technologies, including zinc-finger nucleases, transcription activator-like effector nucleases, and the CRISPR/Cas system, are currently available [2]. The clustered regularly interspaced short palindromic repeat (CRISP)/CRISPR-associated protein (cas) gene is a fiery and accurate genome editing tool, and it has a bacterial adaptive immune system that recognizes and silences foreign nucleic acids, including viruses and plasmids, through small RNA [3]. Genome-editing technology has been widespread in humans [4], animals [5], bacteria [6], and plants [7], and has enabled targeted modification of most crops to accelerate crop improvement. The systems include three types, I, II, and III, which maintain high specificity by guiding the RNA to the target site through base pairing. The Type II CRISPR/Cas9 system from *Streptococcus pyogenes* has been the most commonly utilised gene-editing method to date [8]. PAM is typically a short sequence of 3–5 nucleotides located downstream of the target sequence [9]. Cas endonuclease could cleave invasive DNA with the same original spacer sequence of PAM, resulting in silencing of the exogenous DNA expression and acting as a targeted interfering agent [10]. In addition, Cas9 nuclease could be converted to a nickase that promotes homology-directed repair and undergoes mutagenic activity [11]. The CRISPR/Cas9 system has been applied in many plants, including model crops *Arabidopsis thaliana* [12], rice [13], wheat [14], maize [15], and tobacco [16] to improve abiotic and biotic stress resistance, or to modify vital traits through gene knockout and knock-in. However, *Camellia sinensis* is highly heterozygous due to its self-incompatibility, and its gene-editing transformation system has not yet been established, and gene-editing loci have not yet been tapped.

The selection of gene-editing sites has played a crucial role in improving crop traits. SSR molecular markers, commonly used in crop improvement [17], along with G-quadruplexes, important regulatory elements in gene expression [18,19,20], could potentially influence the type, distribution, and number of gene-editing sites. Among the most effective methods for integrating superior crop traits in one package are molecular breeding and gene editing [21]. SSRs, as the most frequently used molecular markers, are widely distributed in eukaryotic and prokaryotic genomes [22,23,24], with co-dominant inheritance, high polymorphism, repetitiveness, and genome coverage [25,26]. SSR markers have been extensively utilized in germplasm innovation and quality improvement, particularly for screening quality-related markers [17]. Notably, rice, maize, grape, potato, and other crops have witnessed a widespread use of SSR markers [27,28,29,30]. Their characteristic properties and distribution might have an impact on gene-editing loci. G-quadruplexes are nucleic acid secondary structures in DNA and RNA formed by folding of nucleotide sequences rich in guanine bases [18]. The genomic distribution and biological functions of G-quadruplexes have been initially explored in several model plants and important crops, including in *Arabidopsis thaliana*, rice, and wheat [19,20,31,32,33]. G-quadruplexes are extensively distributed in genomic repeat regions such as SSR, ILP, and telomeres [34,35,36], and have been associated with genomic instability as a nucleic acid secondary structure [37] and could affect the efficiency of gene editing mediated by the CRISPR system [38,39,40].

A genome-wide CRISPR screen has greatly promoted the study of the biological function of genes [41]. In recent years, CRISPR screening technology has been applied to plant research, showing great application potential [42]. However, at present, the comprehensive research on CRISPR/Cas9-editing sites in the *Camellia sinensis* genome is still missing. The publication of a high-quality *Camellia sinensis* genome provides conditions for CRISPR/Cas9-editing sites and multi-element association analyses of the genome [43]. In this study, we identified CRISPR/Cas9-editing sites with associated elements at the genome-wide level. Comprehensive analysis of the editing sites and other elements aided in the discovery of specific regions associated with secondary metabolite pathways in *Camellia sinensis*. This study aimed to promote the application of CRISPR/Cas9 technology in *Camellia sinensis* and to provide a reference for the selection of editing loci.

## 2. Results

### 2.1. Frequency and Distribution of CRISPR Loci in the Camellia sinensis Genome

A total of 248,134,838 PAMs were identified in the *Camellia sinensis* genome. These PAMs were predominantly composed of 72,324,043 AGGs, 87,738,084 TGGs, 32,985,030 CGGs, and 55,087,172 GGGs, which accounted for 29.15%, 35.36%, 13.29%, and 22.20% of the whole genome, respectively. Additionally, 509 NGGs were also observed. In particular, 66,665,912 specific PAMs/proto-spacer sequences were present in the genome, including 19,779,444 AGGs, 24,324,960 TGGs, 7,814,091 CGGs, and 14,747,417 GGGs (Table 1). The proportion of TGG was the highest throughout the genome, followed by AGG. The identification of PAM types in the genome and their distribution in chromosomes was consistent with the entire genome, with TGG also exhibiting the highest values in each chromosome (Table 2).

### 2.2. Identification and Characterisation of SSRs in the Camellia sinensis Genome

In this study, 2,938,757,831 bp sequences were examined in the whole genome of *Camellia sinensis*, and a total of 1,352,688 SSR motifs were identified. The results showed that the total length of SSR sequences in the *Camellia sinensis* genome was 39,748,371 bp, which accounted for 1.35% of the total genome length, and the total density and total frequency were 460.29 SSR/Mb versus 13,525.57 bp/Mb, respectively (Table 3). The SSR motifs were classified into eight groups based on the size of the repetitive units, which were mono-nucleotide repeats (MNRs), di-nucleotide repeats (DNRs), tri-nucleotide repeats (TNRs), tetra-nucleotide repeats (TTRs), penta-nucleotide repeats (PNRs), hexa-nucleotide repeats (HNRs), compound (c), and compound* (c*).

**Figure 1:**
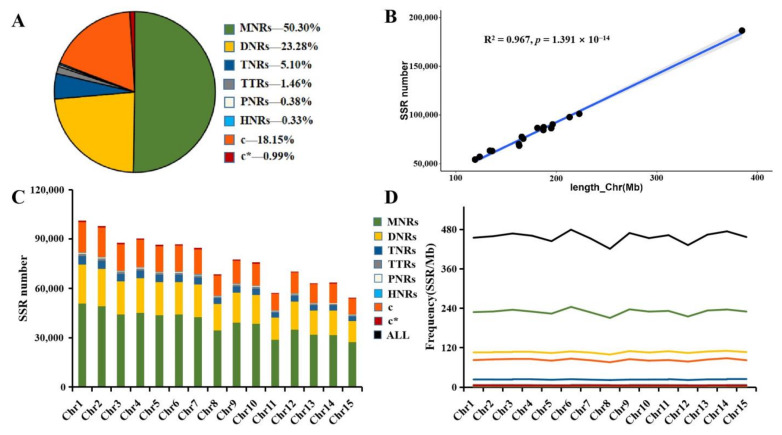
The type, number, and frequency of SSRs in the *Camellia sinensis* genome. (**A**) Types and proportions of SSRs. (**B**) Correlation analysis between chromosome length and the number of SSRs. (**C**) The number of various SSR types in 15 chromosomes. (**D**) The frequency of various SSR types in 15 chromosomes.

Characterisation of the types of repeat units of all identified SSRs revealed that single nucleotide repeats dominated, accounting for more than 50% of the total, followed by dinucleotide, trinucleotide, tetranucleotide, pentanucleotide, hexanucleotide, c, and c* which accounted for 23.28%, 5.10%, 1.46%, 0.38%, 0.33%, 18.15%, and 0.98%, respectively (Figure 1A). With the increase in size of the SSR repeat unit type, the frequency corresponding to the SSRs also appeared to decrease along with it. In addition, SSR motifs identified from the genome sequences were classified according to their location in the genome, and a total of 79.17% of the motifs were found to be located in intergenic regions, and about 20% of the motifs were found to be located in gene regions (Table 4).

**Figure 2:**
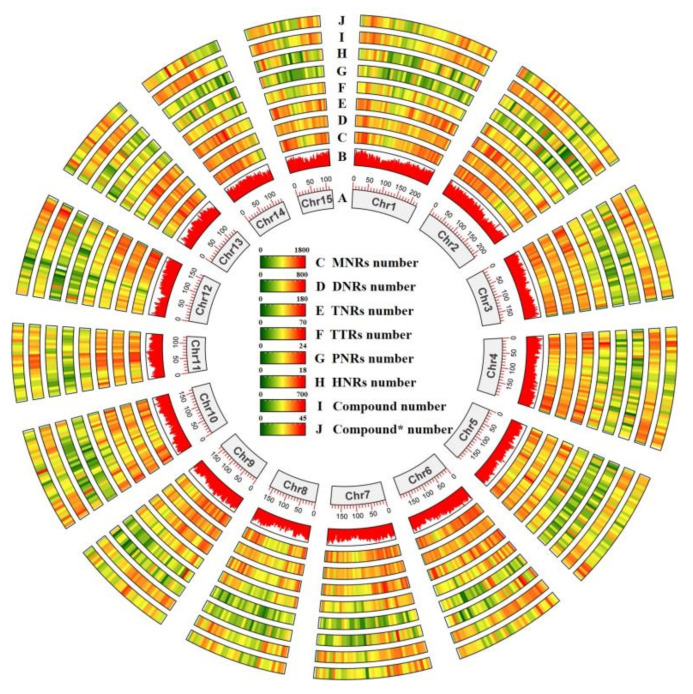
Circos diagram of the density of various SSR types in *Camellia sinensis* genome. (**A**) Chromosome of the *Camellia sinensis* genome. (**B**) Density histogram of SSR occurrences per chromosome. (**C**–**J**) Density heatmap of various SSR types per chromosome. C, MNRs; D, DNRs; E, TNRs; F, TTRs; G, PNRs; H, HNRs; I, Compound; J, Compound *.

Assigning the identified SSR motifs to the corresponding chromosomes revealed a positive correlation between the number of SSRs per chromosome and its length (Figure 1B) (Supplementary Figures S1–S9), with a well-defined linear relationship. It was further divided according to the size of the repeat unit type in each chromosome throughout the genome (Figure 1C) (Table S1). The results indicated that the distribution of single nucleotide repeats per chromosome was similar to the pattern of the whole genome. The percentage of single nucleotide repeats per chromosome was highest, and the number of SSRs per chromosome decreased as the size of the repeating unit increased. The frequency of occurrence of the repeating unit types was delineated (Figure 1D) and was observed to show a similar pattern in each chromosome, with single-base repeats exhibiting the highest frequency. To visualise more clearly the number of repetitive unit sequences in different segments of each chromosome in the *Camellia sinensis* genome, a circle diagram consisting of a heatmap was drawn. The higher the number of repetitive unit sequences, the redder the colour presented in each of its squares, and vice versa, the greener it was (Figure 2). In addition, different types of SSR repeat motifs in the genome were identified. (T/A)n, (AT/TA)n, (AAT/TTA)n, (AAAT/TTTA)n, (AAAAT/TTTTA)n, and (AAAAAT/TTTTTA)n were the most abundant repeat sequence types in each category, respectively (Table S2).

### 2.3. Joint Analysis of CRISPR/Cas9 and Effector Elements

**Figure 3:**
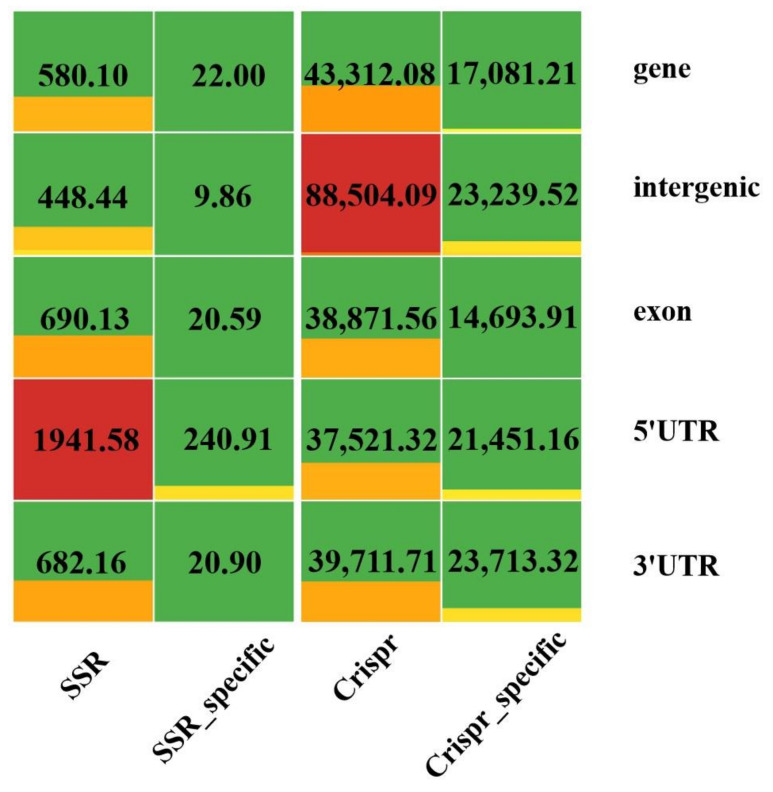
The density (/Mb) of SSRs and CRISPR sites in various feature regions. Green is the background, from light yellow to orange to red represents low to high density, and CRISPR sites uses a larger unit color scale than SSRs.

The densities of characteristic regions and specificity of CRISPR and SSRs were counted for the whole genome, and the total densities of characteristic regions including gene, intergenic, exon, intron, 5′UTR, and 3′UTR SSRs were 4568.83 and 305,399.26, respectively. The densities of specific CRISPR and SSRs were 125,001.61 and 340.93, which accounted for 40.93% and 7.46% of the characterised regions, respectively. Specific CRISPR accounted for 18.59% in the intergenic region and 36.13% in the untranslated region, with smaller differences relative to SSR (Figure 3). Specific SSRs were only 2.89% in the intergenic region and up to 76.79% in the untranslated region.

**Figure 4:**
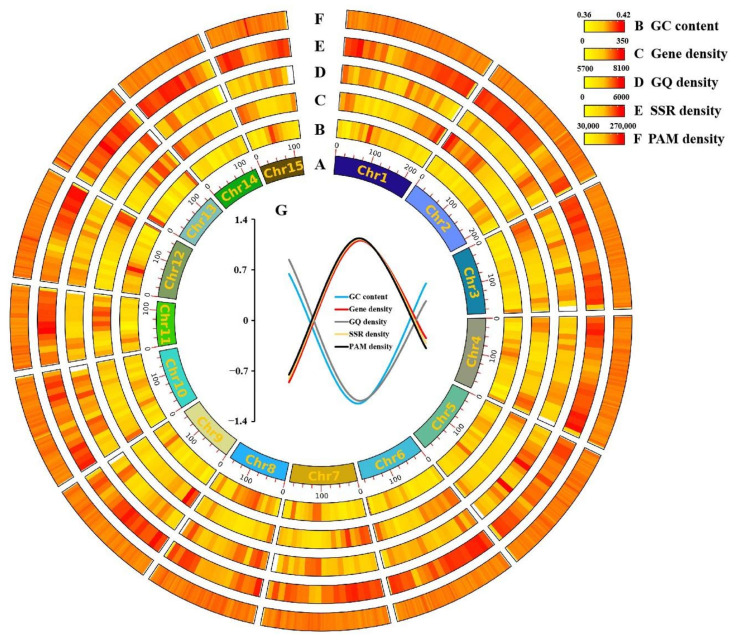
Distribution and analysis of editing sites and multi-elements in *Camellia sinensis*. (**A**) Chromosome of the *Camellia sinensis* genome. (**B**–**F**) Density heatmap of the different element. (**B**) GC content; (**C**) Gene density; (**D**) GQ density; (**E**) SSR density; (**F**) PAM density; (**G**) Standardised fold plot of correlation of various elements in the 120–130 Mbp interval of chromosome 5.

**Figure 5:**
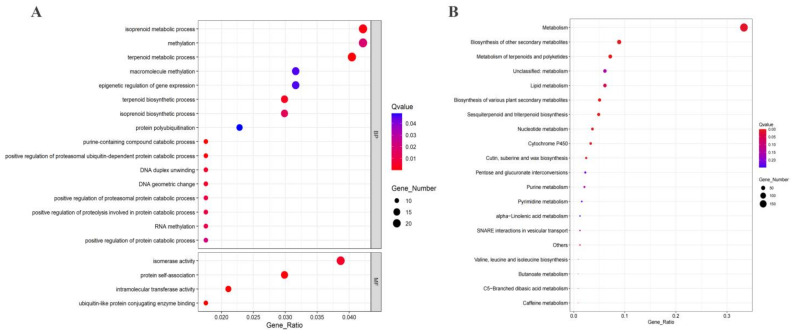
Functional analysis of genes in specific CRISPR-edited regions in 15 chromosomes. (**A**) GO enrichment. (**B**) KEGG enrichment.

In addition, GC content, gene density, GQ density, SSR density, and PAM density were identified in the genome, and mainly SSR and PAM densities were relatively high in the genome. Notably, in the fifth chromosome, it was observed that the GC content, GQ density, and PAM density in these five elements within the 12–13 Mbp range showed opposite trends compared to the gene density and SSR density. The latter two displayed a decrease when the former three densities were high, and this difference was quite significant (Figure 4). The GO and KEGG enrichment analyses were conducted for the specific pattern found in this region (Table 5). The GO enrichment analysis revealed that this region is associated with molecular functions and biological processes. Specifically, terpene biosynthesis processes, genetic regulation of expression, and methylation were found to be significantly enriched as part of the biological processes. The KEGG enrichment analysis indicated that this special region was linked to secondary metabolite biosynthesis and amino acid biosynthesis pathways in the *Camellia sinensis* (Figure 5).

## 3. Discussion

With the development of sequencing technology and the reduction in sequencing costs, a large number of plant genomes have been developed for genetic studies. In recent years, several high-quality genomes have emerged in *Camellia sinensis*, as reference, and the resources of genomes provided data support for the development of gene-editing sites and simple sequence repeats in *Camellia sinensis* [44].

The *Camellia sinensis* genome contained a large number of protospacers and PAMs, which was consistent with the trend that the larger the genome, the more CRISPR/Cas-editing sites would be present in it, compared to *Zea mays* (246,261,552) and *Oryza sativa* (38,923,015) [45]. The identified protospacer regions and PAMs were evenly distributed across the chromosomes. Moreover, the specific PAMs exhibited the same distribution, which suggests a potential for editing *Camellia sinensis* genome regions using the CRISPR/Cas9 system. Across the five PAM types, TGG was the most abundant and CGG was the lowest in number, which followed the same pattern as presented in the chilli and grape genomes [46,47]. NGGs represented a special type of NGG due to the fact that it contained indeterminate base pairs, and these NGGs were mainly found in regions of the genome where the sequence was of low quality. In contrast, gene editing with NGG types was not allowed in the application. Gene-editing systems are becoming increasingly efficient and accurate for targeted gene modification. However, there are still several technical challenges and ethical issues that need to be addressed. Unlike medical and clinical research, plant genome editing does not involve ethical concerns, making it more suitable for applied research [10]. One of the major challenges in this field is applying gene-editing techniques to species that currently lack transformation methods [48]. Additionally, the key genes controlling important agronomic traits are still unknown, posing significant difficulties for genetic improvement through molecular methods [49]. Another important consideration is how to achieve precise gene editing in plants. It is anticipated that with technological advancements, gene-editing systems will eventually be developed for *Camellia sinensis*.

As enriched markers for various studies, whole genome analyses of simple sequence repeats could deepen the knowledge of the genetics and potential functions and have been applied to population structure, varietal identification, construction of genetic maps, and studies of origins, evolution, or domestication history. The SSR density was 460.29 SSR/Mb within the *Camellia sinensis* genome and interestingly, the genome was negatively correlated with SSR density compared to other species [50], unlike the woody plants, where no significant difference was observed in SSR density [51]. The larger number of SSRs will contribute to locating the precise QTLs, excavating the key genes of the traits, and promoting genetic improvement. It is worth noting that CRISPR/Cas9-mediated genome editing may lead to genomic alterations and genomic instability, such as SSR instability [52]. The frequency analysis of SSR motifs in *Camellia sinensis* revealed a negative correlation between the number of repetitions of different SSR motifs and their frequency of occurrence. This finding aligns with the common pattern observed in different species [53,54,55]. Among them, a total of eight different types of motifs were identified in *Camellia sinensis*, and mono- and di-nucleotides were the most abundant SSRs, accounting for 73.58% of the identified whole genome, while the percentage of tri-, tetra-, penta-, hexa-, and composite SSRs was relatively lower, totalling 26.41%. With the increasing number of repetitive units, the proportion of them in the whole genome became lower. The frequency and distribution of SSRs could be explained by the main mechanism of SSR formation. Unique sequences are believed to arise spontaneously through substitutions or insertions, which are then further extended or expanded through the action of transposable elements.

The AT/TA motif was the most prevalent dinucleotide repeat sequence in the *Camellia sinensis* genome. Similarly, T/A, AT/TA, AAT/TTA, AAAT/TTTA, AAAAT/TTTTA, and AAAAAT/TTTTTA were the most frequently occurring motifs in mono-, tri-, tetra-, penta-, and hexa-, respectively. This also means that the most frequent motif in the whole genome might depend on the fact that C or G were less likely to mutate. For instance, AT/TA was the motif with the most dinucleotide repeats in castor [56], wheat was AG/CT [57], tartary buckwheat was AT/TA [58], and potato was AA/TT [30]. Different species might carry their own representative motifs. In addition, the frequency of motifs in non-coding regions was much larger than that in coding regions in the *Camellia sinensis* genome, which might lead to the motifs with more repetitions being richer and more polymorphic than those with comparatively fewer repetitions [59,60]. SSR loci with high polymorphism could be used to evaluate the plant strains and genetic background developed by CRISPR/Cas9 editing. In rice, the CRISPR/Cas9 system (0.8%) resulted in a lower differential SSR ratio between the lines and its recipient, compared to Marker-assisted backcrossing (23.5%) [61]. SSRs located in coding regions, on the other hand, might have implications for genetic studies such as gene regulation. The distribution and frequency of different SSR motifs in different chromosomes indicated that the frequency of SSRs was positively correlated with the size of *Camellia sinensis* chromosomes. The different types of SSR motifs exhibited the same trend in the 15 chromosomes, with their number becoming less and less as the size of the repetitive units increased. Furthermore, the presence of a lower density of SSR motifs in the centromeric region was found in most of the chromosomes in *Camellia sinensis*. This phenomenon might be attributed to the presence of a mitotic region in close proximity to the centromeric region. The mitotic region contained a significant number of transposable factors and highly repetitive sequences, resulting in a lower density of SSRs near the centromeric region of the chromosome [62,63].

GC content, Gene density, GQ density, SSR density, and PAM density were placed together in 15 chromosomes to reveal the relationship between them. For example, in chromosome 5, a sequence was found to be present, and this particular region was always present with high GC content, GQ density, and PAM density and low gene density and SSR density. This might be due to the proximity of this sequence to the mitotic region. The enrichment analysis revealed that this region was related to the biosynthesis of secondary metabolites and amino acids in *Camellia sinensis*. The secondary metabolites and amino acids were essential for the quality and yield of *Camellia sinensis*. Gene editing was closely related to crop breeding as it enabled the elimination of gene loci that did not contribute to desirable crop traits. These changes in gene loci had the potential to enhance yield, quality, and resistance to abiotic stress [64,65]. Gene-editing breeding for target traits using editing loci was expected to produce *Camellia sinensis* varieties with excellent traits that satisfy people’s requirements. In addition, gene editing could also affect gene regulation, for example, by modulating cis-regulatory elements that control transcription, mRNA processing, and translation, and the current techniques to alter gene regulation are focused on promoter regions [66,67]. SSRs, as commonly available molecular markers for crop breeding, could be used for crop improvement by screening for markers associated with traits [68], and the development of SSR markers for special regions could contribute to *Camellia sinensis*’ quality. Previous studies have shown that G-quadruplexes might have a dual effect on the efficiency of *Camellia sinensis* genome editing [39,40]. The study of the relationship between SSR sites, PAM sites, and GQ sites will facilitate the future application of G-quadruplexes in the field of *Camellia sinensis*, for instance, in facilitating the development of gene-editing platforms and heavy metal analyses for food safety.

## 4. Materials and Methods

### 4.1. Identification and Analysis of CRISPR/Cas9-Editing Sites in Camellia sinensis Genome

The CRISPR/Cas9-editing sites in the *Camellia sinensis* genome were identified using the lab’s publicly available perl script (Supplementary Materials crispr_detect.pl), including protospacer, protospacer adjacent motif (PAM), position, and strand. The PAM type was set to NGG and the aimed length of the protospacer was set to 20. The identified PAMs included five types: AGG, TGG, CGG, GGG, and NGG. Considering that some ambiguous nucleotides within the genome are denoted by N, NGG sequences were also recognized.

Specific CRISPR/Cas9-editing sites were defined as CRISPR locus where the protospacer appeared only once in the genome. The PAM in specific CRISPR/Cas9-editing sites were defined as specific PAM. The presence of these sites was analysed using the shell command line.

### 4.2. Identification and Analysis of SSRs in Camellia sinensis Genome

The *Camellia sinensis* genome data and annotation file were downloaded from the *Camellia sinensis* Information Archive database (http://tpia.teaplants.cn/ (accessed on 8 August 2023)) [69]. SSRs in the *Camellia sinensis* genome were identified using MISA, and the parameters were set as definition (unit_size, min_repeats): 1–10 2–6 3–5 4–5 5–5 6–5; interruptions (max_difference_between_2_SSRs): 100; GFF: true [70]. For compound SSR, the interval between two repeats motifs < 100 nt. For compound* SSR, the interval between two repeats motifs < 100 nt, where any two repetitive sequences were unspaced. The correlation between the number of SSRs and each chromosome length was analyzed. The circos diagram of SSR distribution in the genome was created using the Advanced Circos function module of Tbtools v1.108 [71].

For the specific SSR, perl script was processed for the genome annotation file, and the 60 bp sequences before and after exon were intercepted for primer design after removing the CDS column. The primers were screened to remove non-120 bp sequences, primer design was carried out using primer3 [72], the designed primers were screened using perl, the designed primers were subjected to e-PCR, and, finally, the primers verified by e-PCR were screened using the perl script.

### 4.3. Genome-Wide Identification of G-Quadruplexes

The 15 chromosome sequences of the *Camellia sinensis* genome were extracted using the FastaMergeandSplit function module of Tbtools. G-quadruplexes in chromosome were identified by G4Hunter, with window set to 25 and threshold set to 1.2 (https://bioinformatics.ibp.cz/#/analyse/quadruplex (accessed on 5 June 2023)) [73].

### 4.4. Distribution Analysis of Various Sites in Genome Feature Regions

SSR sites were represented by the positions of central bases. The CRISPR-editing sites were represented by the third base of PAM sequences upstream, because the editing sites were located between the third base and the fourth base. Annotation information of feature regions was obtained from genome annotation files, including gene, intergenic, exon, 5′UTR, and 3′UTR. The number and density of all SSRs, specific SSRs, all CRISPR-editing sites and specific CRISPR-editing sites were calculated in the genome feature regions using bedtools [74].

### 4.5. General Landscape and Correlation Analysis of Genome Characteristics

The circos diagram of *Camellia sinensis* genome characteristics was created using the Advanced Circos function module of TBtools, including GC content, gene density, G-quadruplex density, SSR density, and PAM density. The genome characteristic information of the 120–130 Mbp of chromosome 5 was extracted and standardized.

### 4.6. GO and KEGG Analysis

The genome characteristic information of 15 chromosome segments was calculated. Protein function annotation of *Camellia sinensis* was carried out by efficient and accurate eggNOGmapper (http://eggnog-mapper.embl.de/ (accessed on 15 August 2023)) [75]. The annotation result was cleaned using the eggNOG-mapper Helper function module of TBtools. The genes in 15 chromosome segments were focused on as a collection of prospective genes. ClusterProfiler [76] and ggplot2 [77] R packages were used for GO and KEGG enrichment analysis and visualization.

## 5. Conclusions

In the study, we identified 248,134,838 potential editing sites from the *Camellia sinensis* genome and observed five PAM types, of which 66,665,912 were found to be specific and they were present in all structural elements of the gene. Additionally, 1,352,688 SSR motifs were identified in the *Camellia sinensis* genome, and the distribution and frequency of different motifs and repetitive sequences varied across chromosomes. The analysis of editing loci and multiple elements revealed the presence of specific regions in chromosomes associated with secondary metabolites and amino acid biosynthesis pathways. Meanwhile, the editing loci were expected to provide an opportunity for a gene-editing system in *Camellia sinensis*.

## Figures and Tables

**Table 1:** The type and number of PAMs identified in the *Camellia sinensis* genome.

Type	AGG	TGG	CGG	GGG	NGG
All	72,324,043	87,738,084	32,985,030	55,087,172	509
	29.15%	35.36%	13.29%	22.20%	0.0002%
Specific	19,779,444	24,324,960	7,814,091	14,747,417	0
	7.97%	9.80%	3.15%	5.94%	0

**Table 2:** The number of PAMs in the 15 chromosomes of the genome.

Chr	NGG		AGG		TGG		CGG		GGG	
	All	Specific	All	Specific	All	Specific	All	Specific	All	Specific
Chr1	41	0	5,514,034	1,595,139	6,667,511	1,954,182	2,514,745	624,409	4,187,662	1,182,185
Chr2	46	0	5,236,832	1,481,774	6,335,112	1,816,678	2,367,578	574,430	3,974,626	1,096,684
Chr3	38	0	4,576,012	1,261,989	5,563,085	1,556,714	2,040,089	490,275	3,469,647	938,850
Chr4	26	0	4,843,966	1,366,035	5,854,194	1,676,679	2,241,392	548,350	3,690,169	1,015,841
Chr5	52	0	4,843,936	1,290,806	5,856,316	1,585,697	2,233,443	516,796	3,717,723	966,643
Chr6	36	0	4,420,879	1,204,941	5,379,150	1,492,281	2,026,175	471,221	3,367,926	897,928
Chr7	41	0	4,628,598	1,264,538	5,600,957	1,554,148	2,126,672	506,732	3,532,054	945,866
Chr8	30	0	4,126,544	1,174,871	4,951,677	1,435,635	1,935,252	468,605	3,173,432	874,193
Chr9	30	0	4,051,844	1,183,592	4,927,793	1,461,722	1,827,664	460,658	3,068,809	879,000
Chr10	35	0	4,124,404	1,111,348	4,982,046	1,363,741	1,891,552	439,131	3,164,318	831,893
Chr11	21	0	3,046,581	903,785	3,696,195	1,111,851	1,383,057	346,589	2,304,178	664,355
Chr12	36	0	4,051,319	1,084,734	4,902,443	1,325,013	1,870,259	438,932	3,089,787	810,772
Chr13	28	0	3,335,794	932,791	4,066,801	1,152,304	1,505,913	364,932	2,537,034	695,714
Chr14	25	0	3,251,920	943,520	3,969,437	1,167,800	1,459,025	362,874	2,468,483	701,432
Chr15	24	0	2,945,568	804,549	3,581,977	986,519	1,342,645	319,905	2,238,552	599,543
Contig	24	0	9,325,812	2,175,032	11,403,390	2,683,996	4,219,569	880,252	7,102,772	1,646,518

**Table 3:** Summary information of SSRs identified in the *Camellia sinensis* genome.

SSR Type	SSR Number	Total Length (bp)	Average Length (bp)	Frequency (SSR/Mb)	Density (bp/Mb)
MNRs	680,377	8,815,504	12.96	231.52	2999.74
DNRs	314,969	6,035,460	19.16	107.18	2053.75
TNRs	69,020	1,440,693	20.87	23.49	490.24
TTRs	19,775	430,080	21.75	6.73	146.35
PNRs	5166	138,140	26.74	1.76	47.01
HNRs	4495	148,554	33.05	1.53	50.55
c	245,548	21,744,931	88.56	83.56	7399.36
c*	13,338	995,009	74.60	4.54	338.58
All	1,352,688	39,748,371	29.38	460.29	13,525.57

**Table 4:** The number and percentage of SSRs in genome feature regions.

Genome Region		Number	Percentage (%)
Genic	Gene	153,488	11.35
	Exon	14,304	1.03
	5′UTR	5303	0.39
	3′UTR	3818	0.28
Intergenic		1,199,200	88.68

**Table 5:** Segments of *Camellia sinensis* chromosomes with opposite trends in GC content, GQ density, and PAM density to gene density and SSR density.

Chr	Position	GC Content	Gene Number	GQ Number	SSR Number	CRISPR Number
Chr1	70–80 Mbp	0.4088824	68	7308	2762	920,629
Chr2	150–160 Mbp	0.3955052	75	7179	3742	885,913
Chr3	90–100 Mbp	0.3898358	108	6819	3693	862,636
Chr4	50–60 Mbp	0.3934081	94	7273	3975	878,368
Chr5	120–130 Mbp	0.4013279	90	7990	3241	908,802
Chr6	130–140 Mbp	0.3943412	82	6977	3063	877,899
Chr7	100–110 Mbp	0.4008174	73	7523	3143	896,467
Chr8	140–150 Mbp	0.3960673	150	7591	4171	891,675
Chr9	120–130 Mbp	0.4006331	81	7337	3284	898,739
Chr10	50–60 Mbp	0.3972234	76	7337	3636	885,137
Chr11	20–30 Mbp	0.3977763	73	7278	3206	893,461
Chr12	50–60 Mbp	0.4113196	50	7085	2039	932,652
Chr13	80–90 Mbp	0.3833097	148	6746	4664	846,041
Chr14	30–40 Mbp	0.3833575	178	7361	4825	851,661
Chr15	60–70 Mbp	0.3973846	106	7119	3537	886,570

## Data Availability

Data are contained within the article or Supplementary Materials.

## Supplementary Materials

The following supporting information can be downloaded at: https://www.mdpi.com/article/10.3390/ijms242015317/s1.
