# Supplementary material for: CRISPR/Cas9 Editing Sites Identification and Multi-Elements Association Analysis in Camellia sinensis
Source: Int J Mol Sci. 2023 Oct 18;24(20):15317. doi: 10.3390/ijms242015317 (PMC10607008; doi:10.3390/ijms242015317)
Supplement: Supplementary file 1 [file ijms-24-15317-s001.zip › Figures.pdf]

SSR motifs and chromosomes linear correlation plot

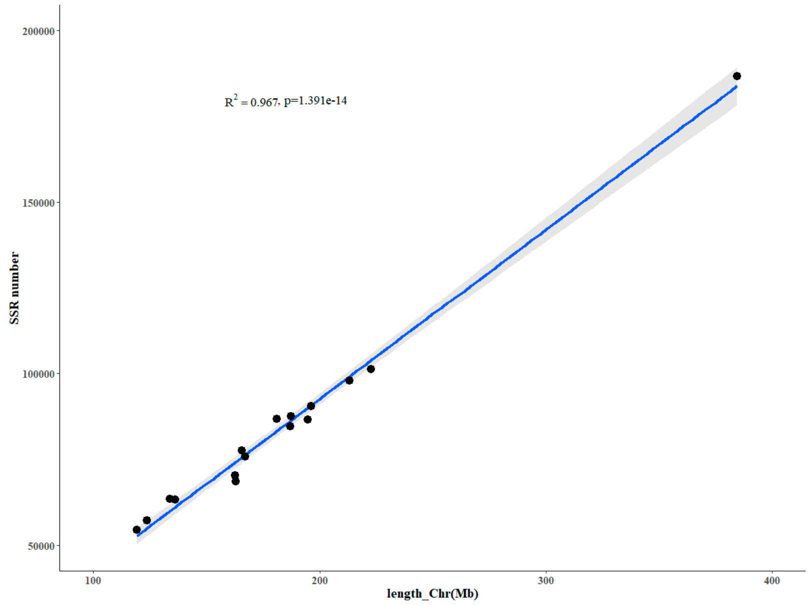

Figure S1.Linear relationship between all identified SSR motifs and chromosome lengths.

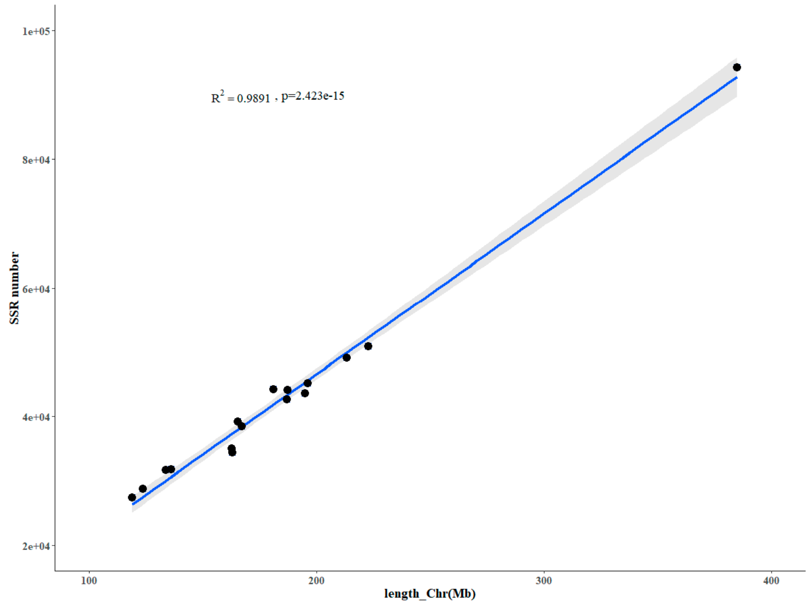

Figure S2.Linear correlation between identified mono-nucleotide repeats motifs and chromosome lengths.

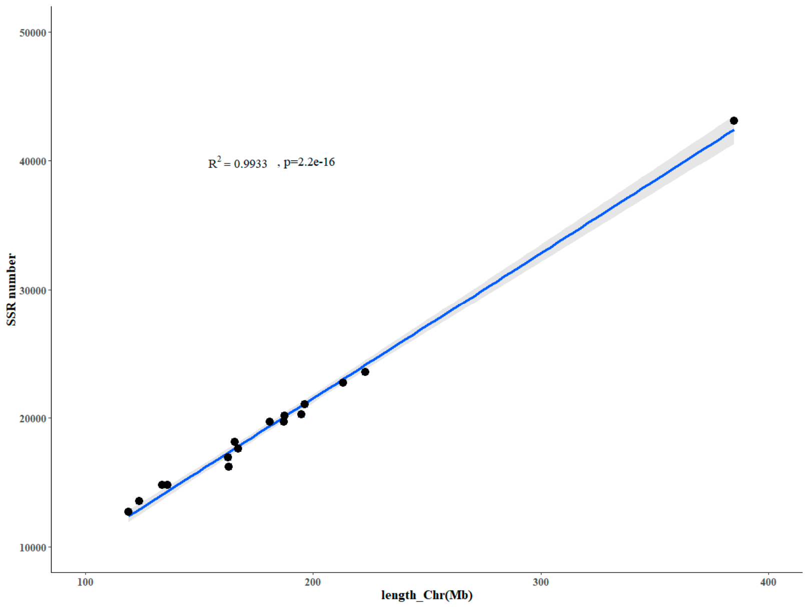

Figure S3.Linear correlation between identified di-nucleotide repeats motifs and chromosome lengths.

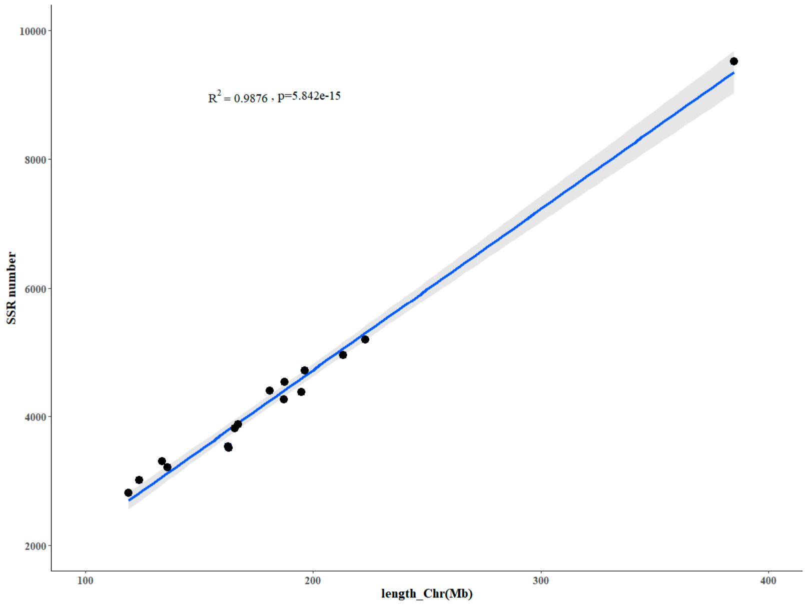

Figure S4.Linear correlation between identified tri-nucleotide repeats motifs and chromosome lengths.

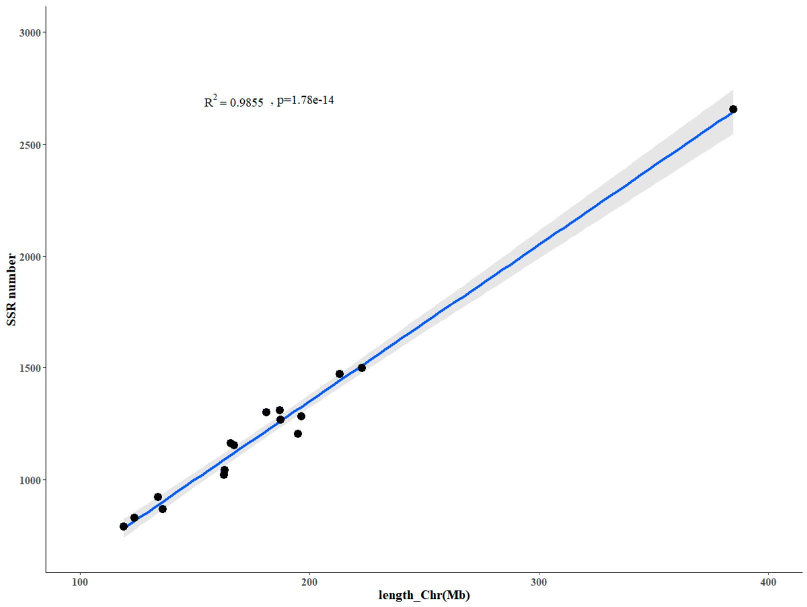

Figure S5.Linear correlation between identified tetra-nucleotide repeats motifs and chromosome lengths.

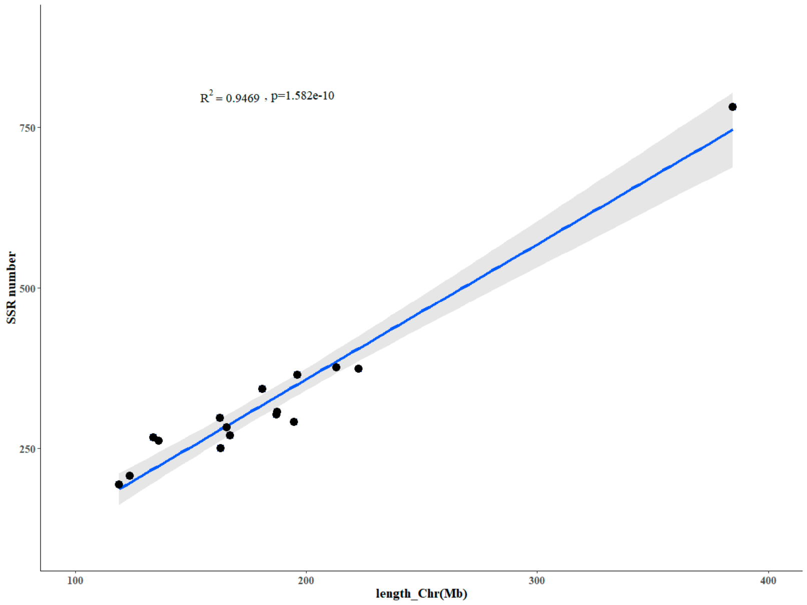

Figure S6.Linear correlation between identified penta-nucleotide repeats motifs and chromosome lengths.

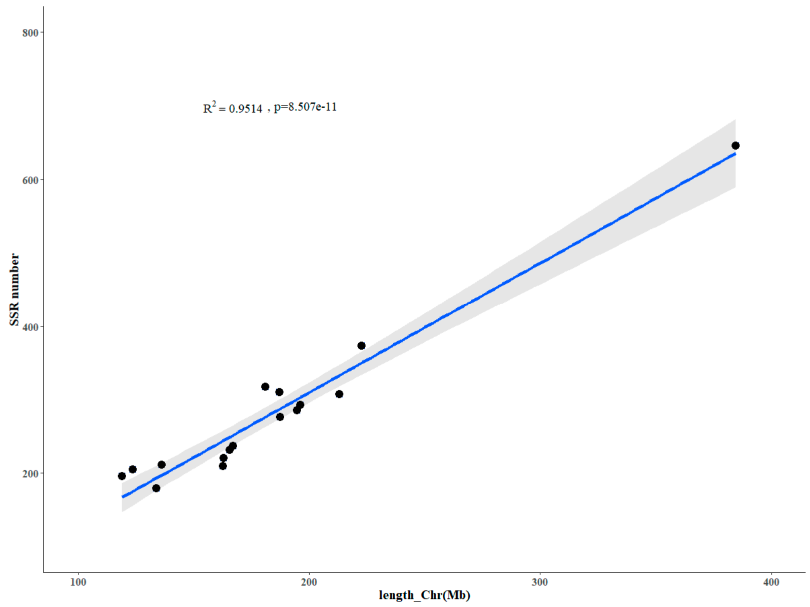

Figure S7.Linear correlation between identified hexa-nucleotide repeats motifs and chromosome lengths.

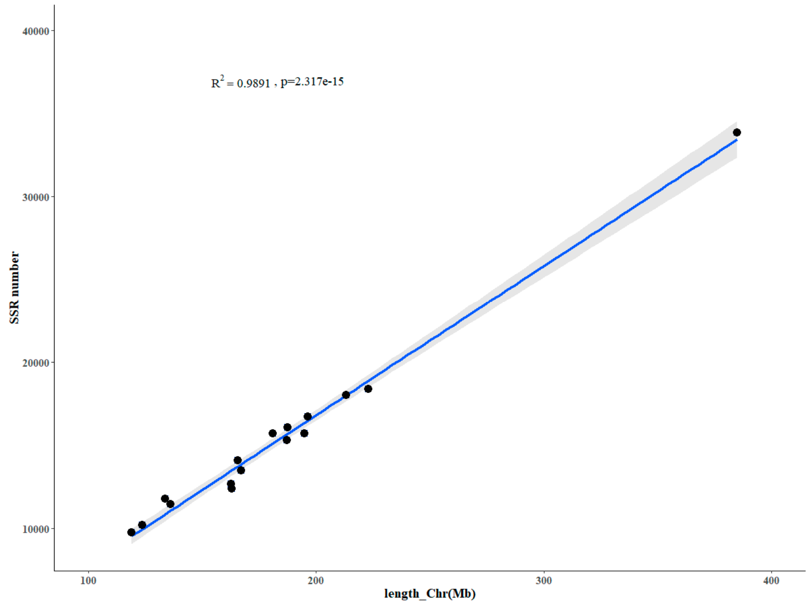

Figure S8.Linear correlation between identified compound motifs and chromosome lengths.

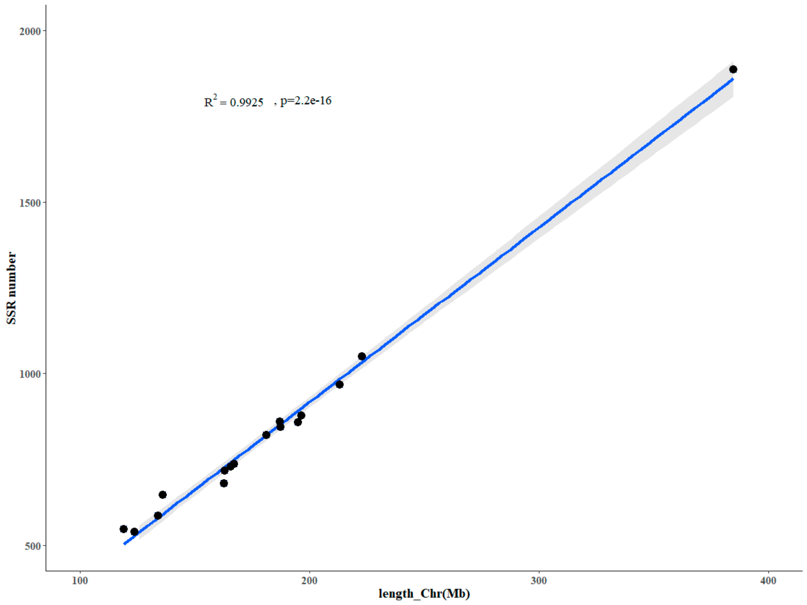

Figure S9.Linear correlation between identified compound* motifs and chromosome lengths.
